# Community structure of gut fungi during different developmental stages of the Chinese white pine beetle (*Dendroctonus armandi*)

**DOI:** 10.1038/srep08411

**Published:** 2015-02-12

**Authors:** Xia Hu, Ming Li, Hui Chen

**Affiliations:** 1 College of Forestry, Northwest A&F University, Yangling, Shaanxi 712100, China; 2 College of Forestry, Fujian Agriculture and Forestry University, Fuzhou, Fujian 350002, China

## Abstract

The Chinese white pine beetle is arguably the most destructive forest insect in the Qinling Mountains in Northern China. Little is known about the structure of the fungal communities associated with *Dendroctonus* *armandi*, even though this wood-boring insect plays important roles in ecosystem and biological invasion processes that result in huge economic losses in pine forests. The aim of this study was to investigate the fungal community structure present in the galleries and guts of *D. armandi* at different developmental stages using PCR-denaturing gradient gel electrophoresis (DGGE). Analysis of PCR-amplified 18S rRNA gene fragments of fungi from the guts of *D*. *armandi* revealed fungal communities of low complexity that differed according to the developmental stage. Yeast of the genus *Candida* and the filamentous fungi *Ophiostoma* predominated in *D. armandi* and its galleries. In particular, *Candida* accounted for 56% of the fungal community in the pupal stage. Characterizing the evolution and content of the intestinal microbial community structure in *D. armandi* may facilitate the development of new pest control strategies.

Bark beetles are among the most ecologically and economically important insects in forests worldwide. Their enormous influence in these ecosystems is enabled by microbial alliances that support their exploitation of trees^1^. Most bark beetle species engage in symbiotic relationships with fungi^2^,^3^ and rely on these fungi to overcome their limited metabolic abilities^4^,^5^,^6^. Bark beetles of the weevil subfamily Scolytinae increase their host-colonizing potential through symbiotic relationships with fungi present in specialized structures called mycangia or on the insect body surface^2^,^7^. Some beetles supplemented their nitrogen-poor phloem diet with fungi (mycophloeophagous) growing in their walls^8^,^9^,^10^. View the knowledge of the bark beetle holobiont discovered to date, it is found that beetles rely on microbes to perform basic life functions and to exploit resources and habitats^1^,^11^,^12^. Thus, associations in bark beetle-fungus systems are of particularly research interest. However, on this topic have been mainly focused on a relatively few North American tree-killing beetles. To better understand these symbioses, studies focusing on interactions between beetles and microbes from other regions of the world are needed.

Bark beetles, particularly *Dendroctonus* species, are serious pests in coniferous forests and cause large economic losses^13^. The Chinese white pine beetle (*Dendroctonus armandi* Tsai and Li, Scolytidae) kills living *Pinus armandi* and has caused serious damage to *P*. *armandi* forests in the Qinling and Bashan Mountains in Northern China since 1954^14^. Various aspects of the biology and physiology of *D. armandi* have been studied, including its niche within the *P*. *armandi* ecosystem^14^, its life cycle^15^, and its symbiotic microbes^16^,^17^. We previously investigated the symbiotic blue stain fungus present in the mycangia of *D. armandi*^16^,^18^,^19^, but studies of the symbiotic intestinal fungal communities of *D. armandi* have been limited.

Fungal communities play important roles in the life histories of bark beetles^7^. The fungi associated with the symbiotic complex may be compared to a ‘root system' or an ‘external stomach' of the insect host due to the ability of these fungi to concentrate large amounts of phloem nutrients^20^. Intimate associations of ascomycetous and basidiomycetous fungi with phytophagous Coleoptera have been reported including not only mutualistic but also commensals and antagonists relationships^21^,^22^. Experimental evidence suggests that certain yeasts in the alimentary canals of bark beetles might be involved in digestive and detoxification processes and in the production of pheromones essential for beetle chemical communication^2^,^23^. Some gut-associated microflora exhibit spatial and geographic variation^24^,^25^, but their potential variation among development stages and between sexes is poorly understood. In general, defining community membership is the first step toward understanding the roles of community members in bark beetle biology and in the functioning of the community itself.

We previously investigated the evolution of gut-associated bacterial communities in the ontogeny of *D*. *armandi*^17^. In the present study, to obtain accurate information on the microbial diversity of *D*. *armandi*, the gut-associated fungal communities of *D*. *armandi* were investigated in the larval, pupal, and adult stages and their galleries in the host *P*. *armandi* using a culture-independent method (i.e., DGGE). We determined which fungal species were consistently associated with the Chinese white pine beetle throughout all developmental stages.

## Results

### Fungal diversity analysis

**Figure 1:**
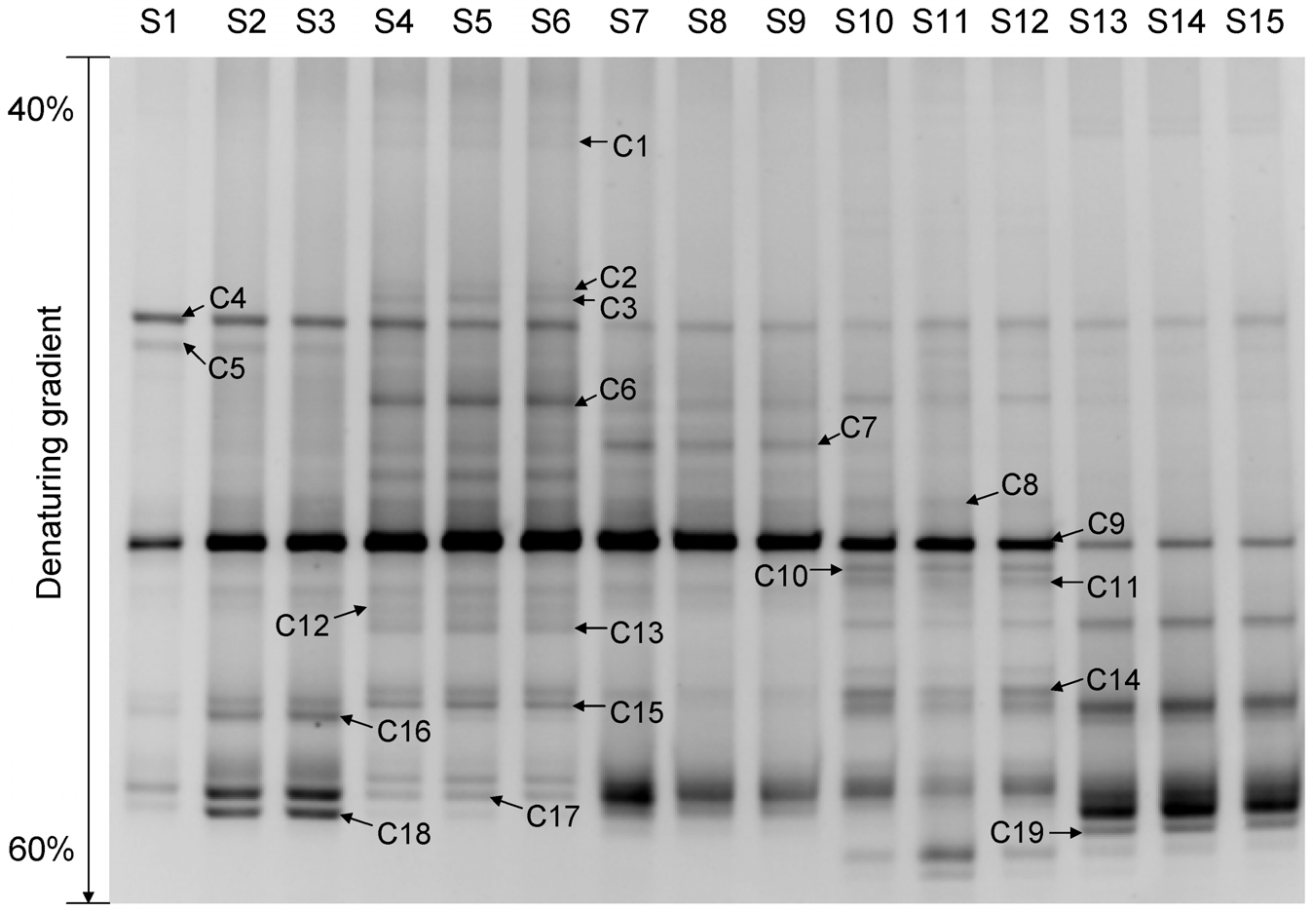
DGGE profiles of nested PCR-amplified 18S rRNA gene fragments of fungi from the galleries and guts of *Dendroctonus armandi* in different development stages. Lanes S1 to S3 correspond to DGGE profiles of three galleries samples, S4 to S6 correspond to DGGE profiles of three *D*. *armandi* larvae samples, S7 to S9 correspond to DGGE profiles of three *D*. *armandi* pupae samples, S10 to S12 correspond to DGGE profiles of three female *D*. *armandi* adults samples, and S13 to S15 correspond to DGGE profiles of three male *D*. *armandi* adults samples. Bands C1 to C19 represent 18S rRNA gene regions of different fungi.

The number, density, and composition of fungal DGGE bands were different at each development stage (Fig. 1). Based on the peak density of bands in the DGGE profile, fungal diversity indices were analyzed to estimate the diversity of the microbial communities, i.e., the value of the Shannon-Wiener index (*H′*) was positively related to the diversity of the fungal community. The richness and *H′* value (2.996725) of the fungi were highest in *D*. *armandi* larval guts (S4–S6), with 21 visible bands in the DGGE profiles, and lowest in the pupal stage (S7–S9), with 12 visible bands (Table 1). ANOVA confirmed that there was a significant difference in fungal diversity (*H′*) between the galleries (S1–S3) and all *D*. *armandi* development stages (*P* < 0.05), except for the pupal stage (*P* > 0.05). The *H′* value was significantly different among all development stages (S4–S15) (*P* < 0.05).

### Phylogenetic analyses and dominant taxa

**Figure 2:**
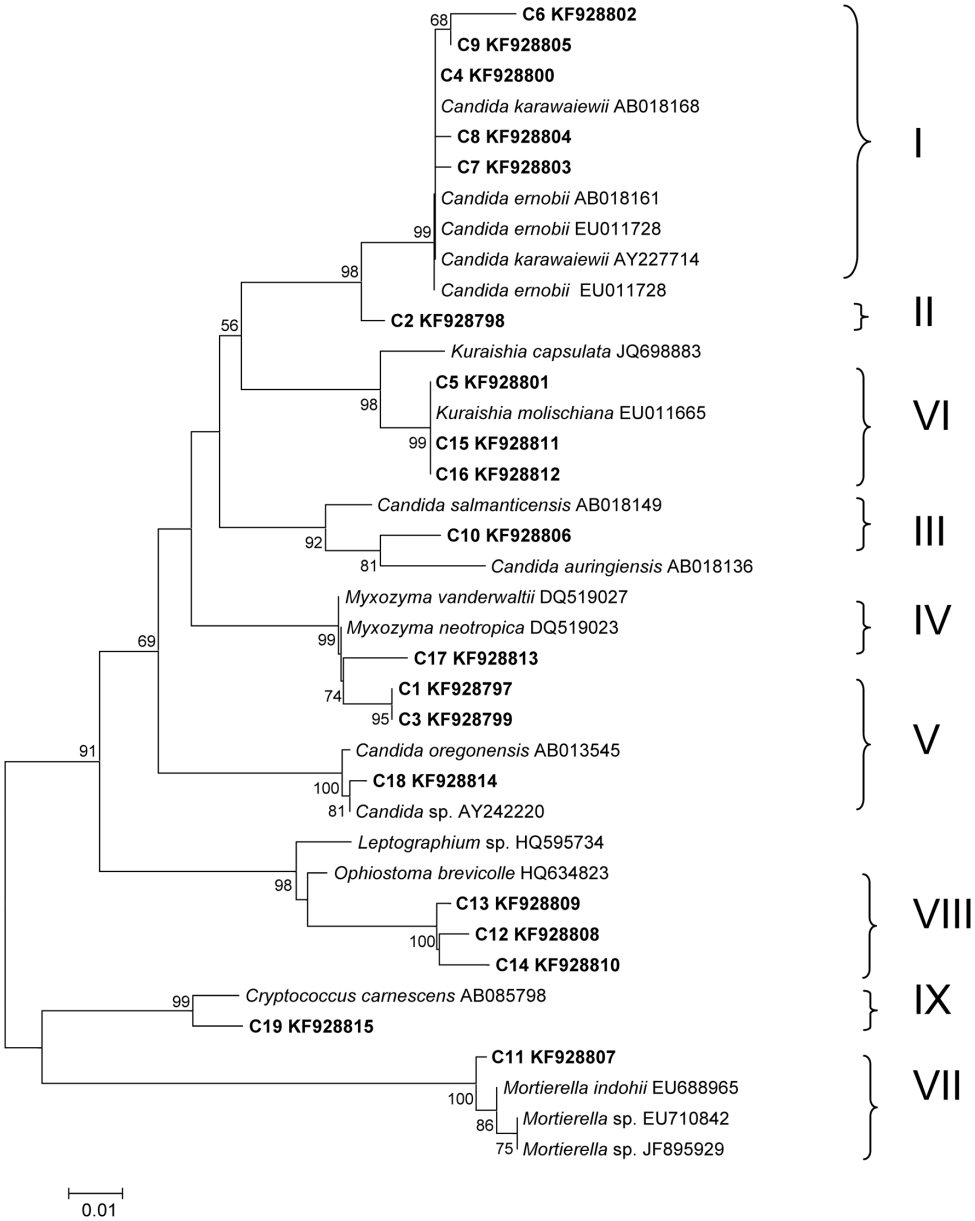
Neighbor-joining phylogenetic tree of the fungal community in the guts of *Dendroctonus armandi* and galleries from their host based on 18S rRNA gene fragments with the model Kimura 2-parameter + G. I–IX represent 9 different OTU_0.03_ clusters obtained using MOTHUR.

In total, 19 different bands were successfully sequenced, and a phylogenetic tree was constructed (Fig. 2). According to the identification results of the 19 bands, it was found that most of the species were related to Ascomycota (C1–C10, C12–C18), with 11 species (C1–C10, C17) belonging to Saccharomycetales. Additional taxa were C11 (Zygomycota, *Mortierella*) and C19 (Basidiomycota, *Cryptococcus*).

The peak density of band C9 was highest in lanes S1–S12, whereas band C19 displayed the highest peak density in lanes S13–S15 (Fig. 1). The phylogenetic analysis indicated that C9 exhibited a high degree of similarity (99% bootstrap support) to *Candida* *ernobii* and *Candida karawaiewii*, and C19 exhibited 99% similarity to *Cryptococcus carnescens*. That is, *Candida* sp. represented the most dominant species in the galleries, larvae, pupae, and female adults, whereas *Cryptococcus carnescens* was the most frequent species among the male adults.

**Figure 3:**
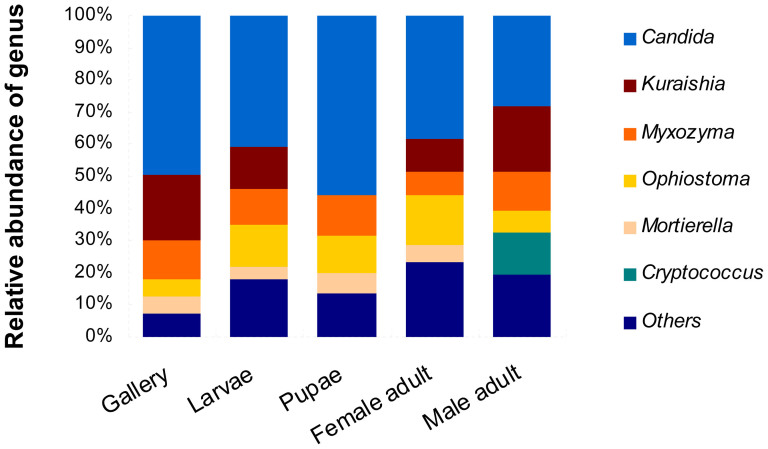
Results of genus-level phylogenetic binning of the *Dendroctonus* *armandi* gut-associated fungal community. Only genera with greater than 1% representation are shown.

Analysis at the genus level identified aboundant sequences matching those of *Candida*, *Kuraishia*, *Myxozyma*, *Ophiostoma*, *Mortierella* and *Cryptococcus* (Fig. 3). The most predominant fungal genus in the galleries and guts of *D*. *armandi* was *Candida* at all development stages. In the pupal stage, the percentage of *Candida* reached 55.9% (Fig. 3). Yeasts of the genus *Kuraishia* were present in all samples except for the pupae. The genus *Mortierella* was present in all samples except for the male adults, whereas *Cryptococcus* was only present in the male adults (Fig. 3).

### Operational Taxonomic Units (OTUs)

The 97% sequence similarity level, corresponding to OTUs_0.03_, was used in the subsequent analyses. Based on the distance matrices of the sequences, nine OTU_0.03_ clusters were obtained and marked as I–IX. The I, IV, and VIII OTU_0.03_ clusters were observed in the fungal communities of all the tested samples, including the galleries and different developmental stages of *D*. *armandi* (Fig. 2). The sequences belonging to clusters I and IV were grouped into the same phylogenetic branch and are closely related to *Candida* (99% bootstrap support) and *Myxozyma* (99% bootstrap support), respectively, cluster VIII is related to *Ophiostoma* (98% bootstrap support) (Fig. 2). In addition, cluster I was the most dominant group among the samples.

**Figure 4:**
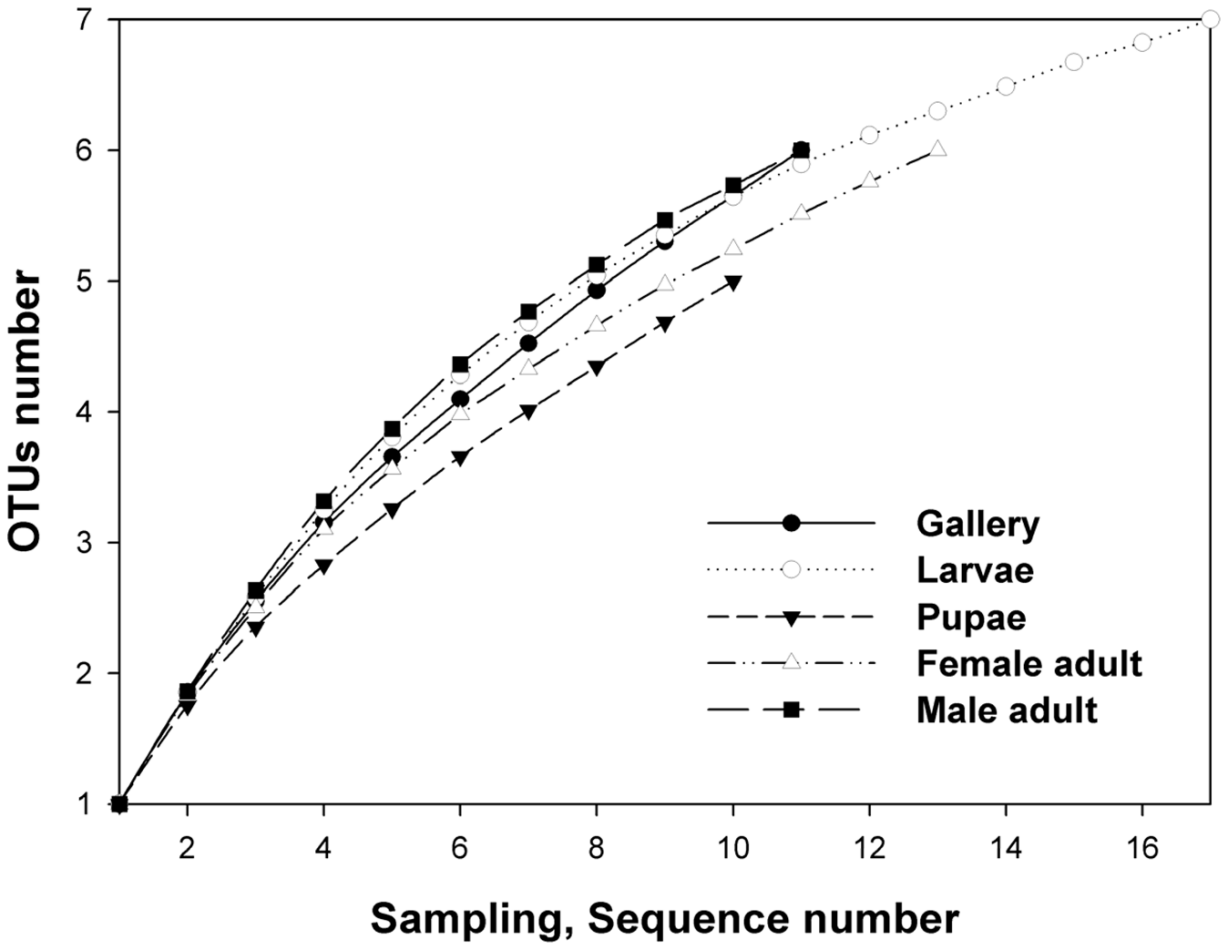
Rarefaction curves of fungal 18S rRNA sequences from the guts of *Dendroctonus armandi* larvae, pupae, female and male adults, and galleries from their host, as calculated using MOTHUR OTU_0.03_. The plot shows the number of new fungal species as a function of the number of clones sequenced.

The Chao index was used to better characterize the relative fungal species richness in the different stages and sexes of *D*. *armandi*. According to the OTU and richness estimation results, the simplest fungal community structure occurred in the pupal gut (Fig. 4), with five OTU_0.03_ clusters (I, IV, V, VII, and VIII) belonging to Ascomycota and Zygomycota (Fig. 2). The larval fungal species richness was the highest among all the developmental stages (S4–S6), with seven OTU_0.03_ clusters (I, II, and IV–VIII) related to Ascomycota and Zygomycota (Fig. 2). The galleries, female and male adult libraries each included six OTU_0.03_ clusters according to the Chao index (Fig. 2). In the guts of female adults, six clusters (I, III, IV, and VI–VIII) were related to Ascomycota and Zygomycota; in male adults, these clusters were I, III–V and VIII–IX, which belong to Ascomycota and Basidiomycota.

## Discussion

Bark beetles are associated with an array of filamentous fungi and yeasts^1^. In our study, gut-associated filamentous fungi and yeasts were surveyed in *D. armandi* beetles and galleries. The present study provides the first insight into the gut fungal diversity of *D. armandi* at different developmental stages and among the two sexes. Overall, the results of the fungal diversity indices constructed based on the peak density of bands in the DGGE profiles were consistent with the richness estimation according to OTUs. The predominant species of the fungal community in *D*. *armandi* formed a group of low complexity, the structure of which differed among the developmental stages. With the exception of fungus-feeding beetles, this low level of fungal complexity is typical of the bark beetle gut discovered to date^22^,^26^,^27^. The diversity of gut-associated fungi in conifer-associated bark beetles is also relatively low, in which only eight fungal species from three genera were identified on the body surfaces of *Dendroctonus ponderosae* larvae, pupae, and adults^28^. Incorporating both culture-based and molecular methods, only 14 yeast species in five genera were identified on the surfaces of *D. ponderosae* Hopkins and *Ips pini* (Say)^27^.

Similarly to previously reported associated bacteria^17^, some fungi were conserved in the galleries and guts of all *D. armandi* stages studied, although not always in the same abundance, including taxa of clusters I (*Candida*) and V (*Myxozyma*) related to yeasts and VIII (*Ophiostoma*) related to the blue stain fungus (Fig. 2). This result indicates that these groups are consistently associated with *D*. *armandi* or their host trees.

Yeasts are commonly associated with bark beetles^27^,^29^ and might be an important nutritional source for the insect host. Furthermore, yeasts might contribute to successful brood development by limiting the numbers of beetles in individual trees, as some yeast can convert the bark beetle's aggregation pheromone trans-verbenol into the anti-aggregation pheromone verbenone^30^. Yeasts that are consistently present in the *D*. *armandi* gut at different developmental stages might influence the development of the insect host.

Ascomycetes of the genus *Ophiostoma*, also known as ‘blue stain fungi', are typically associated with conifers^31^. These fungi produce asexual and sexual spores in slimy masses that attach to insect bodies and are dispersed to new hosts that represent fresh nutrient sources^32^. When bark beetles invade conifers, the fungus taps into the sapwood nitrogen and transports it to the phloem and bark where the beetle larvae feed, increasing the nitrogen content by up to 40%^10^; this is critical for bark beetle development and survival^8^,^10^,^33^. *Ophiostoma* taxa are also the most important filamentous fungal associates of the mountain pine beetle, according to the results of a survey of its body surface, including the mycangia^34^; however, the effects of these fungi on beetles as gut associates are mostly unknown. To further understand the *Ophiostoma-Dendroctonus* relationship, further studies are needed.

The results of the DGGE analyses indicated that Ascomycota species are prevalent in both *D*. *armandi* beetles and their galleries. In the combined diversity indices, Ascomycota belonging to the genus *Candida* was the most abundant taxon. *Candida* is ubiquitous in the environment and responsible for the majority of all fungal infections worldwide^35^. *Candida* species have also been isolated from the guts of other *Dendroctonus* spp.^22^. Suh et al. reported several examples of highly specific associations between certain beetles and yeasts^36^. Our results support this result because certain yeasts were present during the entire life cycle of their beetle hosts. In addition, *Candida* *ernobii* is able to produce lipase, which catalyzes the hydrolysis of long-chain triacylglycerols and plays an important physiological role in several metabolic processes (fat digestion in the gastro-intestinal tract, lipolysis in the adipose tissue, lipolysis of lipoproteins) in the conversion of oils and fats into free fatty acids and partial acylglycerols^37^. *Candida* *ernobii* was the most abundant gut associate and might play similar roles in *D. armandi*. Further experiments are required to verify the roles of yeast, particularly *Candida* spp*.*, in the guts of *D*. *armandi*.

Among the tested developmental stages, the pupal stage was characterized by the simplest fungal structure according to both diversity indices and the Chao index. Low microbial diversity in the pupal gut is common in bark beetles^38^. Energy is required to achieve metamorphosis through the activation of several metabolic processes and morphological changes, even though pupae do not feed. Therefore, the decrease in fungal diversity and increase in *Candida* spp. in pupae observed in this study could be associated with the absence of feeding activity and the necessity for pupae to obtain energy more efficiently by adjusting their microbial community structure.

Attempts have been made to use fungi as biocontrol agents against bark beetle pests in forestry biological control practices^39^. Wegensteiner has presented an overview of the entomopathogenic fungi encountered in European bark beetle galleries^40^. Although few studies have examined the utilization of gut fungi to control bark beetles, gut fungi are candidates for future pest biocontrol strategies. However, detailed knowledge about the dynamic variation, colonization, and modes of transmission of intestinal fungi is required before such a strategy could be successfully implemented.

The information about fungal community structure collected in this study will provide the basis for subsequent studies on the roles of these fungal associates in bark beetle development, ecology, and management. Future studies confirming and quantifying the ability of fungi to degrade tree defenses are required to understand the influences of these fungi on bark beetle biology.

## Methods

### Study sites

The material collection site was the Huoditang Experimental Forest Station of the Northwest Agriculture & Forestry University. The station is located on the southern slope of the middle Qinling Mountains (33°18′–33°28′N, 108°21′–108°39′E), Shaanxi, China. *Pinus armandi*, *Picea wilsonii*, and *Pinus tabulaeformis* are the common tree species distributed throughout most parts of the Qinling Mountains.

### Insect collection and dissection

Larvae, pupae, and female and male adults of *D. armandi* and 0.5 × 2-cm sections of galleries were removed from recently attacked *P. armandi* phloem and placed on ice before transport to the laboratory. **All** *D. armandi* samples were manually obtained directly from galleries of infested pine trees using fine forceps and were then transported to the laboratory in sterile vials containing sterile moist paper. To investigate the influence of sex on gut-associated fungi, female and male adults were separated according to their reproductive organs. For DGGE analysis, 120 beetle samples in each developmental stage and gallery samples were obtained from attacked *P. armandi*. 40 beetle samples in each stage were pooled to be one sample respectively. A total of five samples representing galleries and different beetle developmental stages were obtained. The experiment was repeated twice.

The insect samples were rinsed with sterile water, surface sterilized with 70% ethanol for 3 min, and again rinsed twice with sterile water. After placing in 10 mM sterilized phosphate-buffered saline (138 mM NaCl and 2.7 mM KCl, pH 7.4), the samples were dissected under a stereomicroscope using insect pins to obtain mid-guts and hindguts^41^. Forty guts from each life stage were transferred to 1.5-ml microcentrifuge tubes and then homogenized several times with a plastic pestle in liquid nitrogen, followed by vortexing with 500 μl of Tris-EDTA [10 mM Tris-HCl (pH 8.0), 1 mM EDTA] for 3 min at the speed of 2500 r/min. The homogenate was centrifuged at the speed of 4000 r/min for 15 s to separate the microbial cells from the gut wall tissues and undigested food. The supernatant (containing fungi) was transferred to new tubes for DNA extraction. All procedures were completed in a sterile environment (Biological Air Clean Bench, Suzhou Antai Airtech, Jiangsu, China).

### Fungal DNA extraction

Fungal DNA was extracted using the E.Z.N.A. Fungal DNA Kit (Omega, Biotech, Doraville, GA, USA) according to the manufacturer's directions and stored at −20°C until use.

### Nested PCR

Nested PCR was used to increase the resolution of DGGE^17^. The highly variable region of the fungal 18S rRNA gene was amplified using the primer pairs NS1/EF3 and FF390/FR1-GC^42^. All PCR amplifications were performed in an S1000™ Thermal cycler (Bio-Rad, Hercules, CA, USA) in a final volume of 50 μl containing 25 μl of 2× Taq Master Mix (CoWin Biotech, Beijing, China), 0.8 μl of each primer (10 μM, Invitrogen Trading, Shanghai, China), 1 μl of template, and 22.4 μl of RNase-free water.

The fungal DNA was diluted to 30 ng μl^−1^ and used as the template for first-round PCR, which was performed using the following program: 94°C for 3 min, followed by 30 cycles of 94°C for 50 s, 56°C for 45 s, and 72°C for 50 s and a final extension at 72°C for 5 min. The product of the first-round PCR was diluted 1/200 with ddH_2_O and further employed as the template in the second PCR reaction, which was performed using the following procedure: 94°C for 3 min, followed by 35 cycles of 94°C for 30 s, 58°C for 30 s, and 72°C for 30 s and a final step at 72°C for 5 min. The yield of PCR products and primer specificity were analyzed by 1.2% (w/v) agarose gel electrophoresis and ethidium bromide staining in the presence of the DL2000 DNA marker (Takara Biotechnology, Dalian, China). The obtained PCR products were stored at −20°C until DGGE analysis.

### Denaturing gradient gel electrophoresis (DGGE)

The DCode™ Universal Mutation Detection System (Bio-Rad, Hercules, CA, USA) was used for the DGGE analysis. A 35-μl aliquot of fungal nested-PCR product per sample was loaded onto an 8% (w/v) poly-acrylamide (37.5:1 acrylamide/bio-acrylamide) gel containing a linear denaturing gradient of 40% to 60%, where 100% denaturing acrylamide contained 7 M urea and 40% formamide^43^. The gel was initially electrophoresed at 120 V for 10 min, followed by electrophoresis at 70 V for an additional 10 h at 58°C in 1× TAE buffer [40 mM Tris-acetate (pH 7.4), 20 mM sodium acetate, 1 mM disodium EDTA]. After staining with ethidium bromide solution for 10 minutes, the gel was destained in deionized water for 10 minutes and then photographed under UV light using the Gel Doc™XR System (Bio-Rad, CA, USA).

### DGGE band identification

The dominant DGGE bands were excised from the polyacrylamide gel, and the DNA was eluted using an E.Z.N.A. Mag-Bind Poly-Gel DNA Extraction Kit (Omega, USA). The eluted DNA (1 μl) was re-amplified with primer pair FF390 and FR1 without a GC clamp at the 5′ end. The PCR products were purified using a Gel Extraction Kit (Baitaike Biological Technology, Xi'an, China) and inserted into the pMD18-T vector (pMD18-T Cloning Kit, Takara Biotechnology, Dalian, China). The recombinant plasmids were transformed into *Escherichia coli* (strain DH5a), and positive colonies were identified by blue-white screening. Five positive colonies were randomly selected from each transformation to further confirm the presence of correct inserts via PCR with the primer pair M13-47 and M13-48. The confirmed clones were sequenced (Jinsirui Biotechnology. Nanjing, China).

To classify the intestinal fungi, the obtained sequences were matched to those in the RDP II database^44^ and searched in the NCBI database to select and download reliable and highly similar sequences (≥97% bootstrap support)^45^. The sequences obtained in this study have been submitted to the NCBI database under the accession numbers KF928797–KF928815. The phylogenetic relationships of the intestinal fungi were analyzed by molecular phylogeny. The sequences were aligned using MUSCLE^46^, available in the software MEGA 5.2, and the best model was computed. The phylogenetic trees were constructed using the maximum likelihood and neighbor-joining methods^47^. To calculate the support for each clade, a bootstrap analysis was performed with 1000 replications.

### DGGE band profile analysis

The DGGE band profiles were analyzed using Quantity One software (Bio-Rad, USA) by the following procedure. First, auto frame lanes were selected, and the rolling disk size was adjusted to 5 to minimize the influence of background. Second, the bands were detected, and the parameters were adjusted to acquire the most reliable band pattern; the Gauss model was applied to all lanes. Third, the lane with the most bands was selected for auto-match, and the tolerance was set at 4.00%; the other lanes were matched manually. Finally, the peak density of all lanes was reported for further analysis.

Each band was digitized via auto-detection of the peak density. Based on the transferred data, diversity indices were calculated to investigate the dominant fungal communities and to determine their changes in the galleries, larvae, pupae, and female and male adults of *D. armandi*. Various indices of biodiversity, such as the Shannon-Wiener index (*H′*), richness (*S*), and evenness (*E_H_*), were calculated from the DGGE patterns according to the following equations:
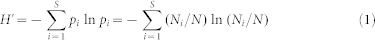

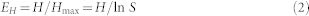


Where *S* is the number of bands in a lane; *Ni* is the peak density of the *i*th band; and *N* is the total peak density of all bands in a lane^48^,^49^,^50^. Significant differences between means were analyzed by the T test in SPSS Version 18.0.

### Operational taxonomic units and richness estimation

The sequences in each phylogenetic tree were formatted as FASTA files and used to construct distance matrices for each library using MOTHUR Version 1.29.0. The distance matrices were used as the input files to define operational taxonomic units (OTUs) on the basis of different similarity distance^51^. This study has been focused on OTUs defined at the ≥97% similarity level (a cutoff of 0.03, OTU_0.03_) to characterize the fungal communities. Although this distance cut-off is arbitrary and could be considered controversial, it has been used in many studies, and it facilitates comparisons with similar studies based on cloning and sequencing^52^,^53^,^54^. Sequences belonging to the same cluster based on the reference of OTU_0.03_ were circumscribed with brackets in the phylogenetic trees and were identified using I-IX for the purposes of clarity. The Chao index was also calculated to measure the absolute value of species richness^55^. Rarefaction curve methodology was used to estimate the relationship between the expected OTU richness and sampling depth^56^,^57^. Finally, rarefaction curves were generated using SigmaPlot Version 10.1.

## Author Contributions

X.H. and H.C. conceived and designed the experiments. X.H. and M.L. performed the experiments and analysed the data. H.C. provided helps in the experiments. X.H., M.L. and H.C. co-wrote the manuscript. All authors discussed the results and commented on the manuscript.

## Figures and Tables

**Table 1:** Richness (*S*), evenness (*E_H_*), and Shannon-Wiener index (*H*′) of intestinal symbiotic fungi identified in galleries and guts of *Dendroctonus armandi* at different developmental stages

Lane	Sample	*S*	*E_H_*	*H*′
S1–S3	Galleries	12	0.966 ± 0.012	2.401 ± 0.031
S4–S6	Larvae	21	0.984 ± 0.002	2.997 ± 0.005
S7–S9	Pupae	12	0.976 ± 0.001	2.419 ± 0.003
S10–S12	Female adults	18	0.981 ± 0.002	2.833 ± 0.005
S13–S15	Male adults	16	0.944 ± 0.003	2.615 ± 0.007

Note: Data are means ± standard error (SE), n = 3.
